# Three-state majority-vote model on small-world networks

**DOI:** 10.1038/s41598-021-03467-6

**Published:** 2022-01-07

**Authors:** Bernardo J. Zubillaga, André L. M. Vilela, Minggang Wang, Ruijin Du, Gaogao Dong, H. Eugene Stanley

**Affiliations:** 1 Center for Polymer Studies and Department of Physics, Boston University, Boston, 02115 USA; 2 Física de Materiais, Universidade de Pernambuco, Recife, Pernambuco 50100-010 Brazil; 3 School of Mathematical Science, Nanjing Normal University, Nanjing, 210042 Jiangsu People’s Republic of China; 4 Department of Mathematics, Nanjing Normal University Taizhou College, Taizhou, 225300 Jiangsu People’s Republic of China; 5 Center of Energy Development and Environmental Protection, Jiangsu University, Zhenjiang, 212013 Jiangsu People’s Republic of China

**Keywords:** Phase transitions and critical phenomena, Complex networks

## Abstract

In this work, we study the opinion dynamics of the three-state majority-vote model on small-world networks of social interactions. In the majority-vote dynamics, an individual adopts the opinion of the majority of its neighbors with probability 1-*q*, and a different opinion with chance *q*, where *q* stands for the noise parameter. The noise *q* acts as a social temperature, inducing dissent among individual opinions. With probability *p*, we rewire the connections of the two-dimensional square lattice network, allowing long-range interactions in the society, thus yielding the small-world property present in many different real-world systems. We investigate the degree distribution, average clustering coefficient and average shortest path length to characterize the topology of the rewired networks of social interactions. By employing Monte Carlo simulations, we investigate the second-order phase transition of the three-state majority-vote dynamics, and obtain the critical noise $q_c$, as well as the standard critical exponents $\beta /\nu$, $\gamma /\nu$, and $1/\nu$ for several values of the rewiring probability *p*. We conclude that the rewiring of the lattice enhances the social order in the system and drives the model to different universality classes from that of the three-state majority-vote model in two-dimensional square lattices.

## Introduction

Over the past few decades, there has been widespread interest in the implementation of tools and methods of statistical mechanics for the study of the behavior and dynamics of social systems; in particular, dynamics of opinion formation^1–21^. Statistical physics studies the complex behavior of macroscopic systems in terms of the basic interaction laws between their many fundamental components. Such complexity shows the emergence of collective effects in the system which are not reducible to the behavior of any individual component or agent. Microscopic models of opinion formation do not intend to mimic human opinion formation dynamics in a completely rigorous way. Instead, they reduce such processes to rather simple interaction laws between the components with very few parameters aiming to understand the possible fundamental mechanisms at play that give rise to macroscopic complexity^17,18,22^.

The dynamics of opinion formation in societies ensue not only from rational thought of individuals, but also from emotional behaviors which are a reflection of the rich social psychology of the influences and interactions between individuals. An example of such a phenomenon is herding, whereby individuals tend to follow the opinion or behavior of their neighbors^16,23–25^. Herding is understood to play a key role in human behavior and in the animal kingdom, from collective behaviors of flocks of birds and schools of fish to riots, strikes, sporting events and opinion formation in social contexts, such as the collective irrational behavior of investors in a financial market who follow the trends of their neighbors and have a propensity to overreact to the arrival of news when buying or selling^26^.

The majority-vote model is a widely studied agent-based model of opinion formation in statistical mechanics^12,13,27–32^. The three-state version of the model assumes that individuals are nodes placed in a social network in any one of three possible states or opinions, interacting with their nearest neighbors, exerting influence on them and being influenced by them in return. In this model, the agent will agree with the majority state of its neighbors with probability $1-q$, or dissent from it with probability *q*, also known as the noise parameter. The noise parameter is a measure of the social temperature of the system, thus inducing the different opinion clusters. The three-state majority-vote model (MVM3) exhibits a second-order phase transition in a square lattice network with critical noise parameter $q_c \approx 0.118$ and with standard critical exponents $\beta /\nu$, $\gamma /\nu$ and $1/\nu$^12–14^.

With the ever-increasing computational power, different variations of the majority-vote model have been considered, including agent differentiation and diffusion, three-state model generalizations and complex noise distributions^15,33,34^. Also, the recent development of complex network science as a statistical-mechanical framework for the study of real networks has also been a contributing factor in the study of complex systems and their dynamics^35–38^.

The original three-state majority-vote model was studied by Tomé on square lattices with periodic boundary conditions^12,13^. Since then, three-state majority-vote dynamics have been studied on non-trivial topologies. For example, the contributions of Melo, Pereira and Moreira extended the study of the dynamics to the topology of random graphs^14^. Vilela et al. delved into the study of the model on scale-free networks, namely Barabási-Albert networks, and three dimensional regular lattices^39^. Both investigations on the three-state majority-vote model conclude that the critical noise required to extinguish consensus increases with the average degree of the network.

In this context, we investigate the dynamics of the three-state majority-vote model on small-world networks. This network enables us to highlight the effects of link disorder, while it keeps the total numbers of nodes and links fixed. Under the small-world link structure, the average degree of the network is a conserved quantity, in contrast with average degree dependence of the majority-vote dynamics investigated on previous works. We focus on exposing the effects of a random link-rewiring scheme on the second-order phase transitions in the system. Additionally, this work also provides a sense of closure to the study of the three-state majority-vote dynamics on complex networks mainly investigated.

The *small-world effect* is the empirical observation that the majority of the pairs of nodes in a great number of real networks appear to be connected by paths of short lengths, even though the sizes of the complex networks are typically very large. More concretely, in a small-world network with *N* nodes, the average distance $\ell$ between two randomly chosen nodes scales as $\ell \sim \log N$^38^. This property of complex networks was given fame and renown thanks to social psychologist Stanley Milgram’s *six degrees of separation* experiment^40–42^. Empirical studies of epidemiology and pandemics reveal that the small-world property became possible with long-range and inexpensive transportation on railroads, airlines and ocean liners^43^. Systems such as the World Wide Web, the Internet and social networks exhibit the small-world property.

The Watts-Strogatz model is a network construction algorithm capable of generating graphs which exhibit the small-world property, with short average path lengths and high clustering coefficients^35,36,38^. The algorithm randomly rewires links in a regular network with probability *p*, which is a tunable parameter, thereby being capable of interpolating between regular lattices and disordered graphs.

In order to explore the small-world effect on the phase transitions of the three-state majority-vote model, we construct the networks with a two-dimensional square lattice subject to a rewiring scheme, whereby we randomly rewire the links in this lattice with probability *p*, in similarity with the Watts-Strogatz model. We use Monte Carlo simulations and finite-size scaling techniques to calculate the critical noise parameter $q_c$, and to obtain the order-disorder phase diagram of the system. We also perform Monte Carlo simulations to estimate the standard critical exponents $\beta /\nu$, $\gamma /\nu$, and $1/\nu$ for the rewiring probabilities *p* considered.

This paper is organized as follows. In Sect. "Square lattices and small‑world networks",we describe the random link-rewiring prescription for the construction of small-world networks starting from a square lattice. We also investigate the main topological features of these networks, as revealed by degree distributions, average clustering coefficients and average shortest path lengths. In Sect. "The model", we describe the model and the dynamics of the social agents, as well as the macroscopic observables of interest to study the phase transitions. In Sect. "Numerical results and discussion", we present the numerical results of the Monte-Carlo simulations, including the phase diagram of the system and finite-size scaling results. In Sect. "Concluding remarks" we present our final remarks and conclusions.

## Square lattices and small-world networks

We consider the three-state majority-vote model in a small-world network, in which each node in the network represents an individual and the links represent nearest-neighbor interactions between pairs of nodes.

**Figure 1:**
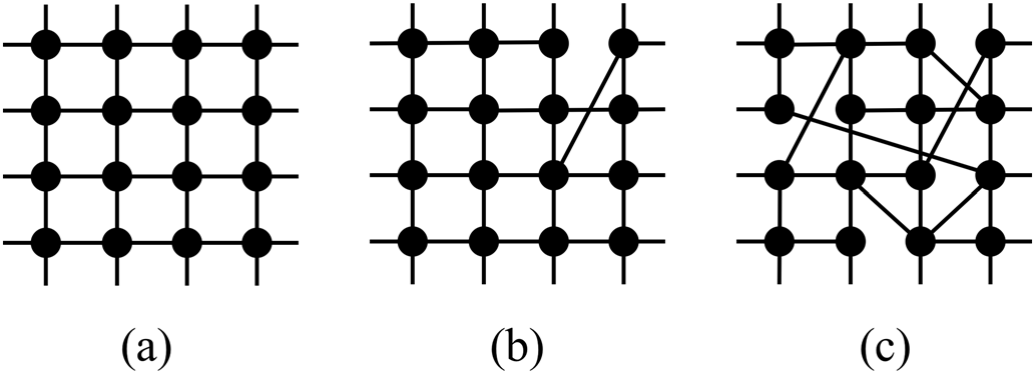
Illustration of the rewiring prescription for the construction of small-world networks. The network starts as a regular lattice shown in (**a**). One obtains (**b**) after the random rewiring of the first link. In (**c**) we illustrate the final result once the rewiring scheme is complete.

In this work, the construction of the structures of small-world networks follows a link-rewiring scheme, illustrated in Fig. 1. We assemble a two-dimensional square lattice with *L* nodes per side and periodic boundary conditions, for a total of $N = L\times L$ agents or individuals. We consider each node in the network sequentially and, for a given node, *i*, the links that connect it to its neighbors to the right and to the bottom are rewired with probability *p* to some other nodes in the network chosen at random. This scheme is forbidden from rewiring the link to another of the original nearest neighbors of node *i* from the initial square lattice. Double connections between the same pairs of nodes are also forbidden. In this way, after repeating the procedure over the *N* nodes, the network is built with the small-world property enabled. It is known that even a minimal number of rewirings, denoted by small values of *p*, is enough to produce the small-world property, shortening the diameter of the network appreciably. In contrast, for $p=1$, one obtains topologies that have maximum disorder. Therefore, *p* is a parameter that controls the topology of the network, interpolating from a square lattice ($p=0$) to a small-world network, to a maximally disordered network ($p=1$), as it increases.

The size of the giant component of the small-world networks built according to the link-rewiring scheme is exactly equal to the size of the whole network for all values of *p*, including $p = 1$. In this context, the rewiring scheme produces networks with only a single component for every value of *p* explored. The investigation of the numbers of components in the networks and of their respective sizes revealed that there was not a single instance of the realization of the disorder, $p \ne 0$, amongst the tens of thousands of networks built, that produced multiple disconnected components or isolated nodes. In other words, the network remains cohesively as a single component as the links are rewired.

**Figure 2:**
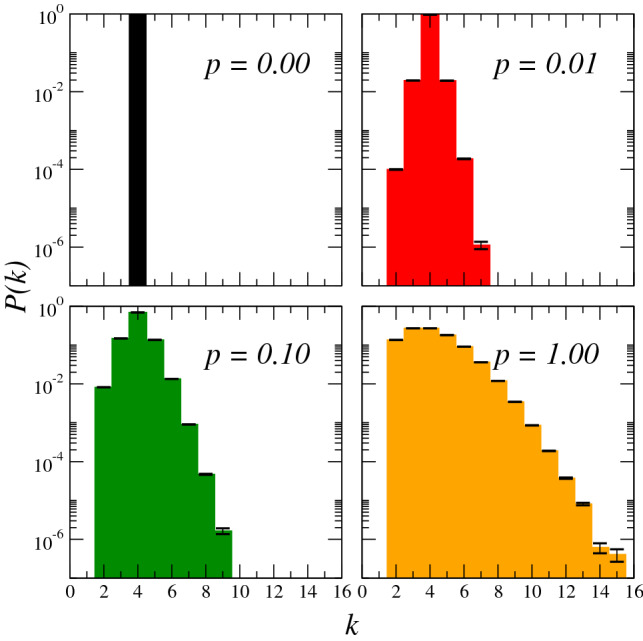
Degree distributions *P(k)* of complex networks built according to the rewiring scheme for four values of the rewiring probability, $p = 0, 0.01, 0.1, 1$ (shown in black, red, green and yellow, respectively). Each sub-figure in the panel corresponds to a fixed value of *p* averaged over a total of 1000 different networks, starting from initial two-dimensional square lattices of size $L = 140$ for a total of $N = 19600$ nodes per network.

The effect of changing the rewiring probability *p* on the topology of the network can be partially illustrated by investigating the degree distribution of the network. Figure 2 presents the degree distribution of the networks built according to the random rewiring prescription described earlier. In the $p=0$ limit, the degree distribution is a Kronecker delta function centered around $k=4$, as expected for a two-dimensional regular lattice with periodic boundary conditions. Increasing the value of *p* has the effect of widening the distribution as some nodes gain connections and others lose them via random rewiring, thereby decreasing the diameter of the network and producing the small-world effect. For $p=1$, we rewire all the links of the original square lattice. Note that the peak of the distribution remains centered around $k=4$. Since no links are being added or removed from the network during the rewiring procedure, the total number of links in the network is conserved. Therefore, the average of the degree distribution is also fixed at $\langle k\rangle =4$, irrespective of *p*.

**Figure 3:**
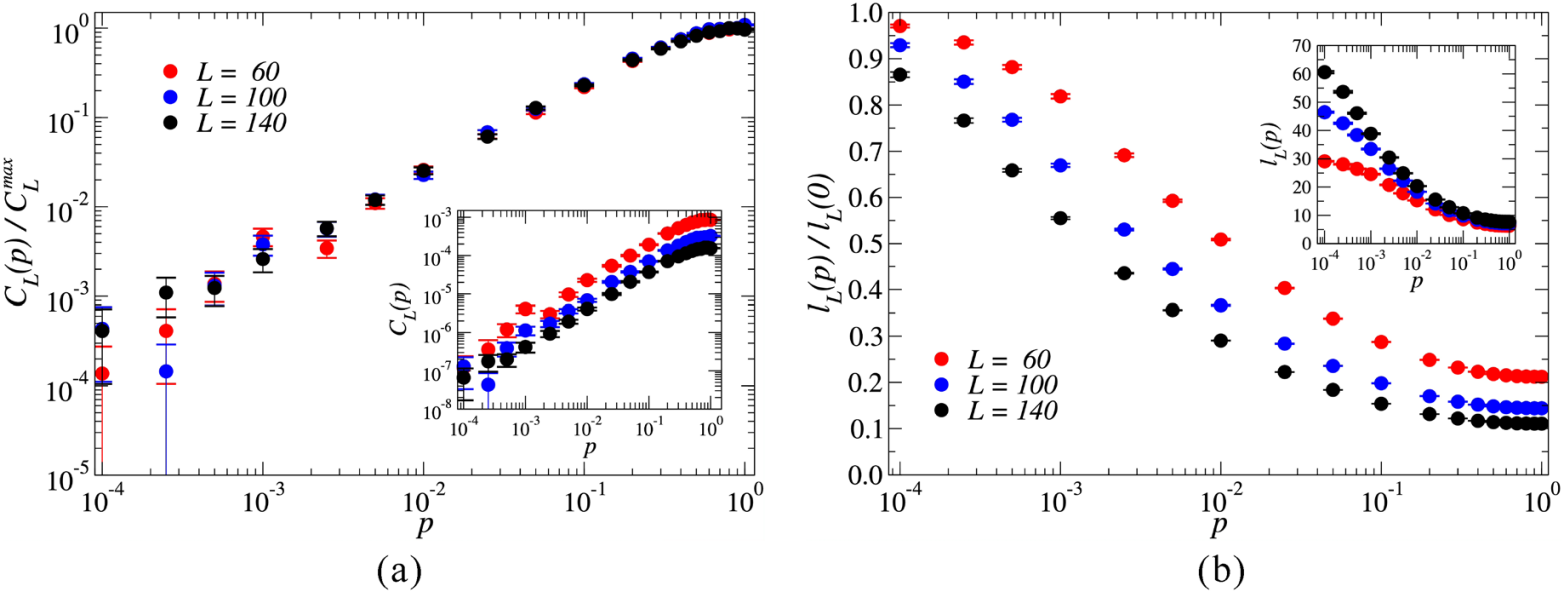
(**a**) Average clustering coefficient $C_L(p)$ of networks with $L=60, 100, 140$, built according to the link-rewiring scheme as a function of *p*. Each data point averages over 1000 different networks. The rates of growth of the $C_L(p)$ seem to be universal and consistent, irrespective of the values of *L* explored in this work, as denoted by the collapse of the curves of $C_L(p)/C_L^{max}$. (**b**) Average shortest path length $\ell _L(p)$ of networks with $L=60, 100, 140$ built according to the square lattice link-rewiring scheme as a function of *p*. Each data point averages over 100 different networks.

In this work, we rewire a square lattice network to yield the small-world structure. Given the differences between the square lattice and the ring-like regular networks in which the Watts-Strogatz algorithm is initiated, a substantive contrast is observed in the behavior of the clustering coefficient. Indeed, using the Watts-Strogatz method, the average clustering coefficient is a monotonically decreasing function of the link-rewiring probability^38^. For the networks explored in this investigation, the local clustering coefficient is zero for all nodes when $p=0$, since the network is a square lattice with periodic boundary conditions. As *p* increases, we expect the increasing disorder to enhance the community structure between the neighbors of an individual node in the network^44^. We illustrate this behavior in Fig. 3a, where we plot the average clustering coefficient $C_L(p)$, which increases with *p* for the values of *L* explored in this work. The curves are normalized relative to the maximum average clustering coefficient measured $C_{L}^{max} = \max _{p} C_L(p)$ for each value of *L*. The average clustering coefficient $C_L(p)$ is the arithmetic average of all the local clustering coefficients. Each data point averages over 1000 different networks built in accordance with the square lattice link-rewiring scheme.

In Fig. 3b, we show the effect of the square lattice link-rewiring scheme on the average shortest path length $\ell _L (p)$. The curves are normalized relative to $\ell _L(0)$. For a given pair of nodes (*i*, *j*) in the network, the trajectory with minimal path lengths ($d_{ij}$) connecting *i* and *j* is considered. The average shortest path length $\ell _L(p)$ is computed as the arithmetic average of all such shortest path lengths $d_{ij}$ between all of the $N(N-1)/2$ pairs of nodes (*i*, *j*) in the network. As expected, the networks are susceptible to the increase of *p*, showing that even a small fraction of links rewired is sufficient to effectively shrink the network’s diameter. Indeed, rewiring only $1\%$ of the links in the networks with $L=140$, for instance, is enough to drop $\ell$ to approximately $30\%$ of the value it would have in the case of the square lattice. These networks settle, at maximum rewiring $p = 1$, at a final average shortest path length that is roughly $10\%$ that of the square lattice $p = 0$ case. This shows the emergence of the small-world effect in the networks considered in this work as topological disorder is increased by random link-rewiring.

## The model

With the network of social interactions successfully built, we execute the dynamics of the three-state majority-vote model. In this model, each agent’s opinion is represented by a node and it assumes any one of three available states $\sigma \in \{1,2,3\}$ at any instant in time. These states represent three possible opinions on a given social subject or referendum. For example, in a financial context, such states could represent a desire to purchase a unit of an asset, to sell a unit of an asset or to remain neutral/inactive. It may also represent a citizen’s vote in an election where there are three available political options: left-wing, right-wing or centre.

The update of an opinion follows a probabilistic prescription. The state of each individual tends to follow the local majority, i.e., there is a tendency to agree with the opinion of the majority of its nearest neighbors with probability $1-q$, or to dissent from it with probability *q*. Consider agent *i* in the event of a single majority state of its neighbors. The agent *i* may adopt it with probability $1-q$, whilst each of the two local minority opinions may be adopted by *i* with probability *q*/2. In the event of a tie between two local majority states, agent *i* shall assume any of those two opinions with probability $(1-q)/2$ each, and the opinion of the local minority with probability *q*.

Let $k_{i, \sigma }$ represent the number of nearest neighbors of agent *i* that find themselves in state $\sigma \in \{1,2,3\}$, with $k_{i, 1}+k_{i, 2}+k_{i, 3}=k_i$, where $k_i$ stands for the degree of agent *i*. For square lattice networks with nearest-neighbor interactions, $p = 0$ and $k_i = 4$ for all $i \le N$. The aforementioned rules for the update of the state of agent *i* following the three-state majority-vote dynamics with noise *q*, for any network, can be summarized thus:

1 $$\begin{aligned}&P(1|k_{i,1}>k_{i,2}; k_{i,3})= 1-q, \\ &P(1|k_{i,1}=k_{i,2}>k_{i,3})=(1-q)/2,\\ &P(1|k_{i,1}<k_{i,2}=k_{i,3})=q,\\ &P(1|k_{i,1} ; k_{i,2}<k_{i,3})=q/2,\\ &{P(1|k_{i,1} = k_{i,2} = k_{i,3})= 1/3.} \end{aligned}$$

The probabilities for the remaining two states (2 and 3) follow easily from the symmetry operations of the $C_{3\nu }$ group. It is worth noticing that the condition $P(1|\{k_i\})+P(2|\{k_i\})+P(3|\{k_i\})=1$ holds for any configuration $\{k_i\}\equiv \{k_{i,1};k_{i,2};k_{i,3}\}$ of the connected neighbors, as it should, for the update probabilities to be conserved. The model is defined for values of the noise parameter in the range $0\le q\le 2/3$, since it may increase from zero to the amount corresponding to the situation in which each opinion is equally probable $1-q = q/2$.

### Numerical quantities

We quantify the degree of opinion order of these social systems in terms of an order parameter. Following the analogy with magnetic systems, we shall adopt the magnitude of the average magnetization *m*, defined in analogy with the three-state Potts model^45^. The instantaneous magnetization consists in a vector with components: $m_\sigma$ for $\sigma \in \{1,2,3\}$. Its magnitude is thus given by $m=\sqrt{m_1^2 +m_2^2 + m_3^2}$, and its components are defined as:

2 $$\begin{aligned} m_\sigma = \sqrt{\frac{3}{2}} \left[ \frac{N_{\sigma }}{N} - \frac{1}{3}\right] , \end{aligned}$$

where $N_{\sigma }$ is the number of agents in state $\sigma$ and $N=L^2$ is the total number of nodes in the small-world network. With this definition, it can be shown that the components of the magnetization are not independent of one another. In fact, it holds that $m_{1}+m_{2}+m_{3}=0$. Also, the magnitude of the instantaneous magnetization of the system becomes $m=0$ in the fully disordered phase, i.e, when all three states are equally populated ($N_{\sigma }/N = 1/3$). The fully ordered phase exhibits $m=1$, where only one state is populated, e.g., $N_1/N = 1$ and the remaining two are vacant $N_2/N = N_3/ N = 0$.

The order parameter proper, given by the average magnetization of the system, is therefore computed as

3 $$\begin{aligned} M_L (q,p) = \langle \langle m\rangle _t\rangle _c , \end{aligned}$$

where $\langle ...\rangle _t$ denotes time averages taken in the stationary regime of the system, and $\langle ...\rangle _c$ denotes configurational averages.

We shall also be interested in quantifying the magnitude of the fluctuations of the order parameter of the system. For this purpose, we define the magnetic susceptibility of the system as a measure of the variance of the order parameter in analogy with Ising-like magnetic systems

4 $$\begin{aligned} \chi _L (q,p) = L^2 [\langle \langle m^2\rangle _t\rangle _c - \langle \langle m\rangle _t\rangle _c^2]. \end{aligned}$$

As is the case near the critical points of magnetic systems, we expect the susceptibility to peak and attain its maximum value in the vicinity of $q_c$. Indeed, in the thermodynamic limit $L \rightarrow \infty$, the magnetic susceptibility would exhibit singular behavior manifested as a divergence at $q_c$.

We also evaluate the fourth-order Binder cumulant as a measure of the kurtosis of the order parameter $M_L (q,p)$ of the system, defined as follows

5 $$\begin{aligned} U_L(q,p) = 1 - \frac{\langle \langle m^4\rangle _t\rangle _c}{3{\langle \langle m^2\rangle _t\rangle _c}^2}. \end{aligned}$$

This quantity tends to 2/3 deep in the ordered phase of the system and it decreases to zero well into the disordered phase. It can be shown that, for sufficiently large system sizes, the Binder cumulants are rather insensitive to the system size, and their curves cross each other at the same point $q_c$, regardless of *L*, thus providing an estimate of the critical point in the thermodynamic limit ($L \rightarrow \infty$).

It has been shown numerically that the three-state majority-vote model exhibits a second-order phase transition in a square lattice network ($p=0$), with a critical noise parameter of $q_c \approx 0.118$ and with critical exponent values given by $\beta /\nu =0.134\pm 0.005$, $\gamma /\nu =1.74\pm 0.02$, in accordance with the three-state Potts model, for which $\beta /\nu =2/15$, $\gamma /\nu =26/15$ and $\nu =5/6$^12–14^.

## Numerical results and discussion

We perform Monte Carlo simulations on small-world networks built on underlying two-dimensional square lattices with linear sizes ranging from $L=40$ to 140. In this work, a Monte Carlo Step (MCS) is defined as unit of time in our simulations, corresponding to a total of $N=L^2$ attempts of changing the opinion states of the agents in the network. We prepare the initial configuration by setting the opinion state of all agents to $\sigma = 1$. To allow the system to thermalize and reach a steady state in a given simulation, we skip the first $5\times 10^4$ MCSs, after which we evaluate time averages over a window of another $2\times 10^5$ MCSs. We repeat the simulations for up to 200 different samples for the calculation of the configurational averages.

**Figure 4:**
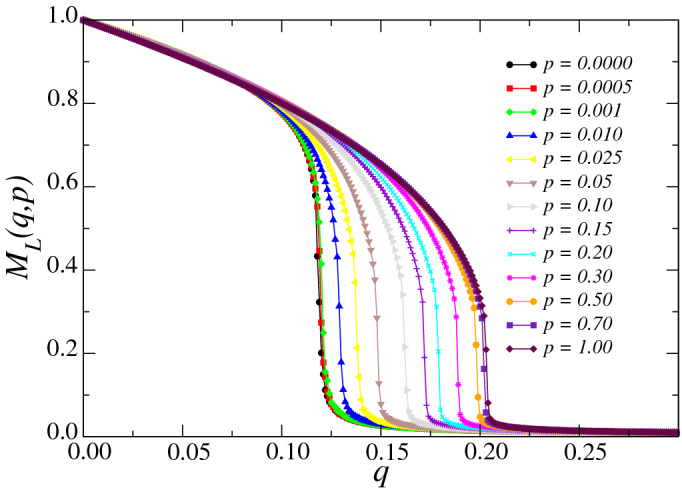
Average magnetization $M_L (q,p)$ as a function of the noise parameter *q* for several values of the rewiring probability *p* and $L=140$. For each value of *p*, $M_L \sim O(1)$ for $q < q_c(L)$ and $M_L \sim 0$ for $q > q_c(L)$. The error bars are smaller than the symbol size, and the lines are just a guide to the eyes.

In Fig. 4, we show the dependence of the average magnetization $M_L$ for several values of the rewiring probability *p* and $L=140$. The curves of the order parameter $M_L$ suggest the presence of spontaneous magnetization for low *q*, where $M_L \sim O(1)$. We note that $M_L \sim 0$ when increasing *q* above some critical value, which depends on *p*. Figure 4 also exhibits the existence of second-order phase transitions occurring between ordered and a disordered states. From this figure, we conclude that the presence of long range interactions promote consensus for the three-state majority vote model, since the noise value that induces a vanishing magnetization increases with the rewiring probability *p*.

**Figure 5:**
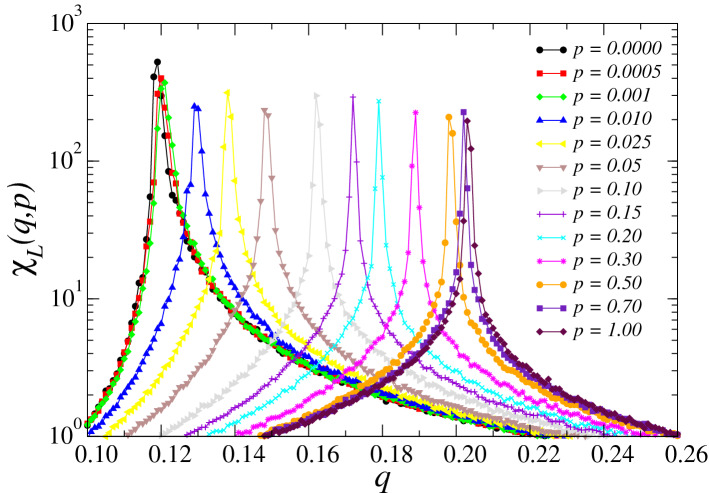
Magnetic susceptibility $\chi _L (q,p)$ as a function of the noise parameter *q* for several values of the rewiring probability *p* and $L=140$. The locations of the maximum values of $\chi _L$ indicate the approximate critical noise $q_c$ parameters for each value of *p*. The error bars are smaller than the symbol size, and the lines are just a guide to the eyes.

In Fig. 5, we show the curve of the magnetic susceptibility $\chi _L$ obtained from Eq. (4) versus the noise parameter *q*. The data from $\chi _L$ provide further evidence for the phase transitions on the three-state social system. The maxima of these curves indicates the approximate location of the critical noises $q_c$ for each value of the rewiring probability *p* for a system of size $L = 140$. We observe that these critical noise values depend on the system size *L*; thus, we name them pseudocritical points, denoted by $q_c(L)$. Further analysis on the size dependence provide a precise evaluation for $q_c = q_c(L)$ with $L \rightarrow \infty$, which does not depend on the system size.

**Figure 6:**
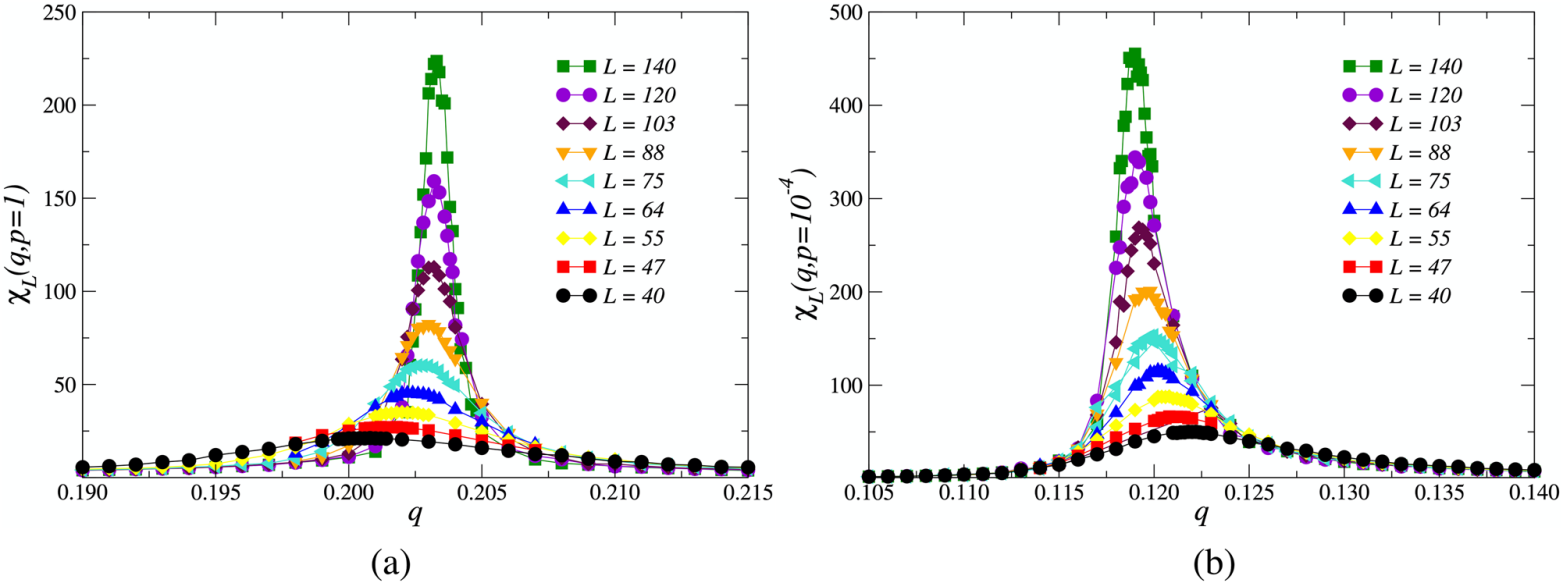
Magnetic susceptibility peaks for different system sizes and (**a**) $p=1$ and (**b**) $p=10^{-4}$. For large values of *p*, the pseudocritical points $q_c(L)$ shift to higher values of *q* with increasing system size *L*. For low values of *p*, the the peaks of the susceptibility, and thus the pseudocritical points, shift to lower values of *q* with increasing *L*.

We observe a substantial topological phenomenon for the scaling law of the magnetic susceptibility of the Eq. (8). The locations of the magnetic susceptibility peaks, denoted by the pseudocritical points $q_c(L)$, tend to move horizontally as we vary *p*. For higher values of the rewiring probability *p*, the locations $q_c(L)$ of the peaks shift to higher values of *q* as *L* increases, eventually settling at the actual critical noise $q_c$ in the thermodynamic limit $L\rightarrow \infty$. However, for low values of the rewiring probability *p*, the locations $q_c(L)$ of the peaks shift to lower values of *q* as *L* increases. In Fig. 6, we illustrate this effect on the peaks of $\chi _L(q, p)$ for two values of *p*.

The reason behind this interesting effect is tied to the nature of the small-world network construction process used, i.e., the link-rewiring scheme. The small-world effect increases with increasing *p*, and the diameter of the network shrinks with increasing probability of link rewiring. This effect becomes stronger still and more pronounced with increasing linear system size *L*, since, effectively, the diameter of the network (and the average geodesic distance between nodes) decreases relative to the size of the network *N* as *L* increases. Therefore, as shown in Fig. 6a, this causes the pseudocritical points $q_c(L)$ to shift to higher values of *q* when the topological disorder of the network *p* is large, making the ordered phase of the system more stable with increasing system size. The counterpart to this situation has a similar explanation. When *p* is small, the small-world property of the network is weak. Thus, the square lattice structure dominates the critical behavior of the social network. In this context, the pseudocritical points decrease with the increase of the linear system size *L* as expected. Therefore, as Fig. 6b illustrates, the susceptibility peaks shift to smaller values of *q* with increasing system size. In Fig. 9c we show the line fits to the data for $\ln \chi _L$ at $q_c$ versus $\ln L$, where the slopes for each line provide an estimate for the critical exponent $\gamma /\nu$ of the model.

**Figure 7:**
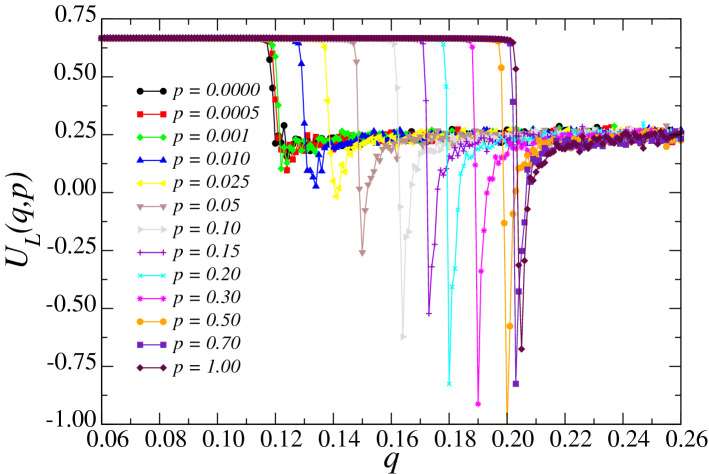
Binder fourth-order cumulant’s $U_L (q,p)$ dependence on the noise parameter *q* for several values of the rewiring probability *p* and $L=140$. $U_L (q,p)$ moves to the right as the rewiring probability *p* increases, in agreement with $M_L (q,p)$ and $\chi _L (q,p)$. The error bars are smaller than the symbol size, and the lines are just a guide to the eyes.

In Fig. 7 we illustrate the Binder cumulant $U_L$ of the system for several values of the rewiring probability *p*. The curves of fourth-order Binder cumulants $U_L$ similarly suggest the existence of a phase transition, given their visible drop from the value of $U_L=2/3$ around the locations of the critical noise values previously observed in Figs. 4 and 5. This qualitative picture of the behavior of the pseudocritical points $q_c(L)$ indicates that the critical noise parameter $q_c$ is an increasing function of the rewiring probability of the small-world networks *p*.

**Figure 8:**
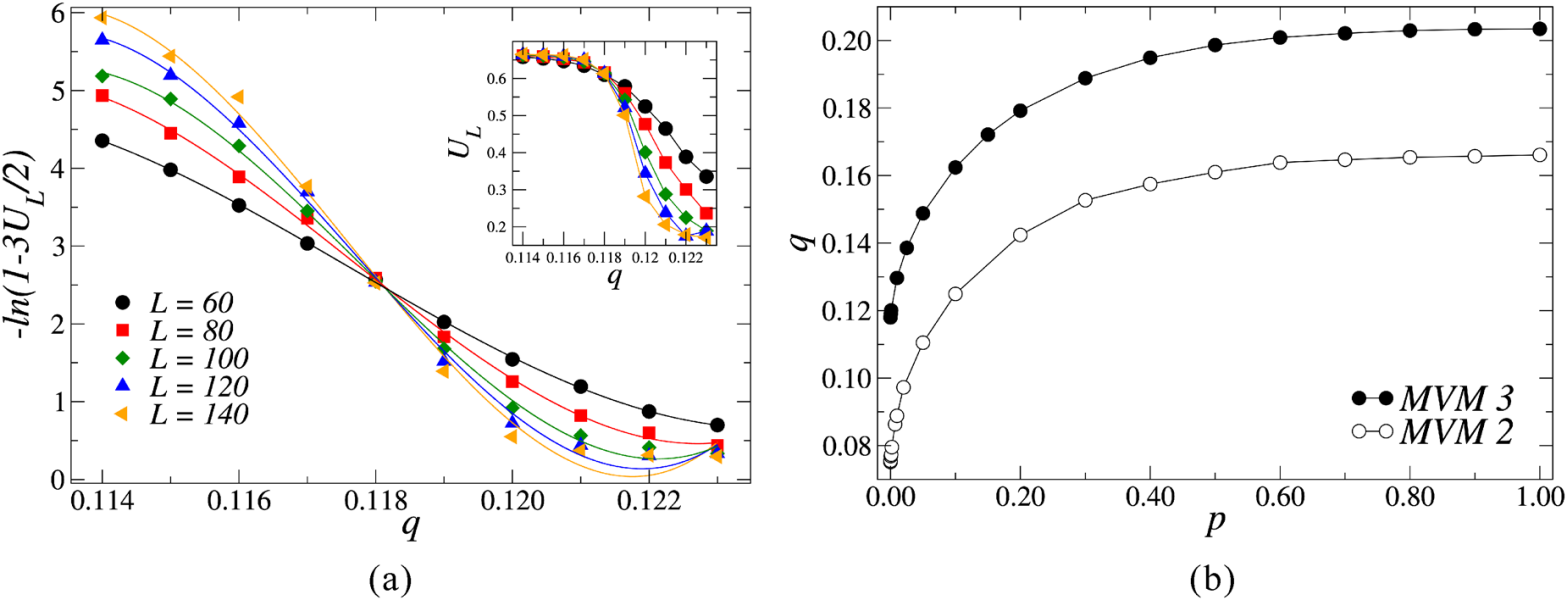
(**a**) Fourth-order Binder cumulant $U_L$ as a function of the noise parameter *q* for $L=60,80,100,120$ and 140. The lines are cubic polynomial fits to the data, yielding for $p=0.0001$ a critical noise $q_c=0.1181\pm 0.0002$. In the inset we show an overview for $U_L$ and the lines are guides for the eye. (**b**) Phase diagram of the three-state (two-state) majority-vote model in small-world networks (open symbols). The points represent the critical noise parameters $q_c$ as function of the rewiring probability *p*. The error bars are smaller than the symbols, and the lines are guides to the eye.

The Binder cumulant defined in Eq. (5) does not depend on the system size *L* at the critical point of the system $q_c$ for any value of the rewiring probability *p*. We explore this property to estimate the critical noise parameter for each *p* in the thermodynamic limit, $q_c(p)$, as the points where the fourth-order Binder cumulants $U_L$ for different sizes *L* intercept each other. In Fig. 8a, we present the Binder cumulant versus the noise parameter *q* for the rewiring probability $p = 0.0001$, and $L=60,80,100,120$, and 140. Here, we estimate $q_c=0.1181\pm 0.0002$. We repeat the evaluation of $q_c(p)$ for several values of the rewiring probability *p*, thus enabling the construction of the phase diagram of Fig. 8b. In this phase diagram, we exhibit the order-disorder transition for the three-state majority-vote model (closed symbols) and the phase diagram for the two-state version (open symbols)^34,44^, for comparison purposes. The ordered phase lives below the curves for each system, for which there is spontaneous magnetization and symmetry-breaking. Conversely, the region of points that lie above the curve correspond to the disordered and symmetric phase of the system, where the average magnetization is zero. The phase diagram clearly presents the observation made earlier that the critical noise parameter $q_c$ is a monotonically increasing function of the rewiring probability *p*, with asymptotic behavior in the critical noise parameter in the $q\rightarrow 0$ and $q\rightarrow 2/3$ limits.

The previous set of results suggests that increasing the rewiring probability, thereby reducing the diameter and average path distance of the network, enhances the long range ordering effect on the opinions. Moreover, it is clear that, for small values of *p*, a small increase in the rewiring probability increases the critical noise substantially and, for large values of *p*, it quickly saturates as the networks become more random and the decrease of the average path length is no longer appreciable. Therefore, increasing the randomness in the network topology makes the ordered phase more resilient against thermal noise.

We remark that the small-world topology developed in this investigation allows us to highlight the contribution of long-range interactions to the robustness of the ordered phase in the three-state majority-vote dynamics for a fixed average degree $\langle k\rangle = 4$. Complementary studies suggest that the order in this particular model can also be improved by increasing the average connectivity $\langle k\rangle$ of an agent^14,39^.

### Phase transitions on small-world networks

To obtain the three standard critical exponents that characterize the phase transitions of the model, we note that in the region of criticality $q - q_c \simeq 0$, the pseudocritical noise $q_c(L)$, the average magnetization $M_L$, the magnetic susceptibility $\chi _L$ and the Binder cumulant $U_L$ satisfy the following finite-size scaling relations:

6 $$\begin{aligned} q_c (L)&= q_c + bL^{-1/\nu }, \end{aligned}$$

7 $$\begin{aligned} M_L (q,p)&= L^{-\beta /\nu }\widetilde{M}(\varepsilon L^{1/\nu }), \end{aligned}$$

8 $$\begin{aligned} \chi _L (q,p)&= L^{\gamma /\nu }\widetilde{\chi }(\varepsilon L^{1/\nu }), \end{aligned}$$

9 $$\begin{aligned} U_L (q,p)&= \widetilde{U}(\varepsilon L^{1/\nu }), \end{aligned}$$

where *b* is a constant, $\varepsilon =q-q_c$ is the distance to the critical noise, and $\widetilde{M}$, $\widetilde{\chi }$ and $\widetilde{U}$ are universal scaling functions that depend on the scaled variable $x=\varepsilon L^{1/\nu }$.

**Figure 9:**
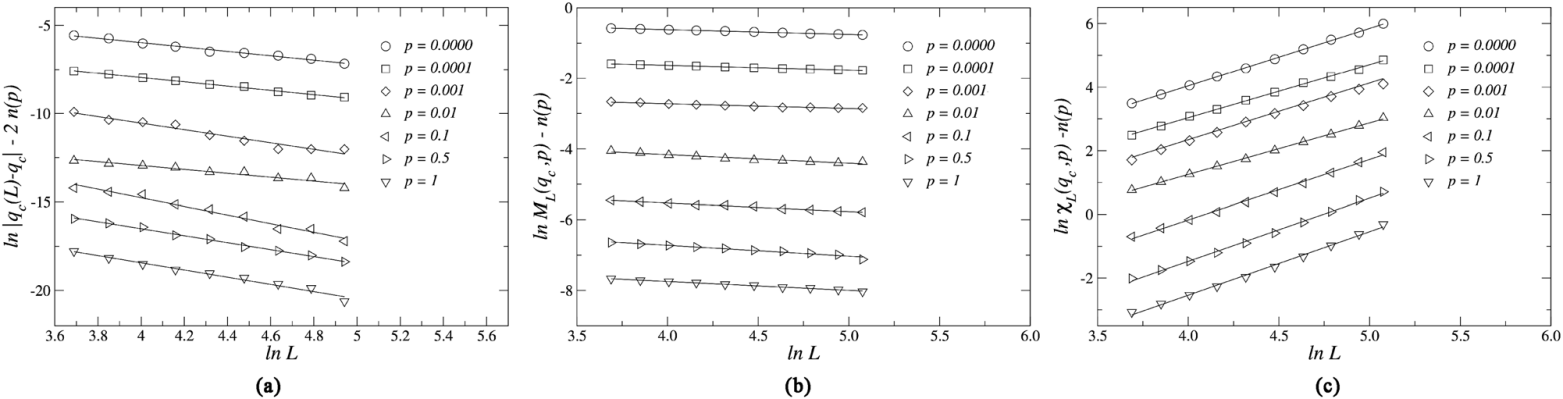
Plot of (**a**) the distance $\ln |q_c(L)-q_c|$ between the pseudocritical points and the critical point $q_c$, (**b**) the magnitude of the magnetization $\ln M_L$, and (**c**) the magnetic susceptibility $\ln \chi _L$ as functions of the of $\ln L$ for different rewiring probabilities *p*. The slopes of (**a**–**c**) yield estimates for the critical exponents $1/\nu$, $\beta /\nu$, and $\gamma /\nu$, respectively. The function *n*(*p*), defined in Table 1 is used to separate the curves corresponding to different values of *p* for the sake of visual clarity.

Using Eq. (6), we estimate the critical exponent $1/\nu$ exploring the pseudocritical noise dependence on the system size *L*. In Fig. 9a we plot the logarithm of the distance between the pseudocritical point and the critical noise $|q_c (L)-q_c|$ versus the logarithm of $L$. The slopes of the linear fits to the data provide numerical estimates for the critical exponent $1/\nu$ for each rewiring probability *p*. In this figure, we use the function *n*(*p*), defined in Table 1 to separate the curves corresponding to different values of *p* for the sake of visual clarity. We perform a similar analysis using Eqs. (7) and (8) to obtain the critical exponents $\beta /\nu$ and $\gamma /\nu$, associated to the order parameter and to the magnetic susceptibility, respectively. In Fig. 9b,c we show the log-log plots of $\ln M_L$ and $\ln \chi _L$ as a function of the logarithm of the system size $\ln L$ for different rewiring probabilities *p*, where the slopes of each line fits to the scaled data, and provide numerical estimates for the critical exponents of the model.

**Table 1:** Definition of the function *n*(*p*).

*p*	0	$10^{-4}$	$10^{-3}$	$10^{-2}$	$10^{-1}$	$5\times 10^{-1}$	1
*n*(*p*)	0	1	2	3	4	5	6

**Table 2:** Table of critical noise parameters $q_c$, their correspondent critical exponents $\beta /\nu$, $\gamma /\nu$, and $1/\nu$, and the effective dimension of the system $d_{eff}$ for the three-state majority-vote model on small-world networks for different rewiring probabilities *p*.

*p*	$q_c$	$\beta /\nu$	$\gamma /\nu$	$1/\nu$	$d_{eff}$
0	0.1180(2)	0.136(3)	1.76(2)	1.25(5)	2.03(2)
0.0001	0.1181(2)	0.152(7)	1.70(3)	1.28(2)	2.00(3)
0.001	0.1201(3)	0.16(1)	1.69(4)	1.28(1)	2.01(5)
0.01	0.1297(2)	0.24(2)	1.63(5)	1.05(5)	2.12(8)
0.1	0.1624(1)	0.24(1)	1.89(4)	1.58(1)	2.37(6)
0.5	0.1986(1)	0.31(2)	1.91(3)	1.97(5)	2.53(8)
1	0.2035(3)	0.25(1)	1.90(3)	2.04(8)	2.42(6)

In Table 2 we provide the estimates for the critical exponents $1/\nu$, $\beta /\nu$ and $\gamma /\nu$ of the three-state majority-vote model on small-world networks. We also present the value of the effective dimension of the system for each value of *p*, calculated as $d_{eff} = 2\beta /\nu +\gamma /\nu$, in accordance with the hyperscaling relation for critical systems for *p* in the small-world regime. The case for $p=0$ corresponds to the regular square lattice, where the numerical results for both the critical noise parameter and the critical exponents agree with the numerical calculations provided by previous investigations, consistent with the universality class of the three-state Potts model on the square lattice^12,13,45^. As seen in the table, the values of $d_{eff}$ suggest that the effective dimensions of the small-world system are close, as expected, to $d=2$.

We remark that when *p* increases away from the small-world region, where $p \ge 0.1$, the system goes through different topological regimes, as discussed in Fig. 6, and the values of the critical exponents are quite challenging to calculate. However, it is well known that the effective dimension for complex networks does not necessarily equal the space dimension on which they are inserted^34,46–49^.

**Figure 10:**
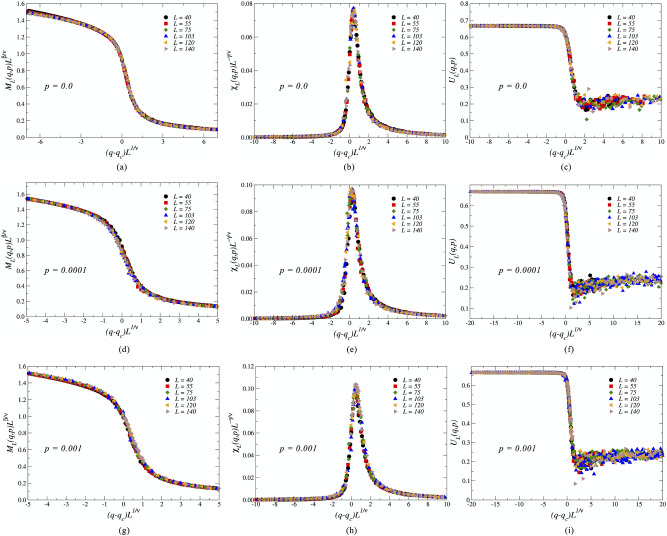
Data collapse into universal scaling functions for the average magnetization $M_L$, the magnetic susceptibility $\chi _L$, and for the fourth-order Binder cumulant $U_L$ for regular square lattices, i. e., $p=0$, and for small-world interactions with rewiring probability $p = 10^{-4}$ and $p = 10^{-3}$.

The estimations of the values of these critical exponents serve as a guideline for plotting the scaling functions $M_L (q,p) L^{\beta /\nu }$, $\chi _L (q,p) L^{-\gamma /\nu }$, and $U_L (q,p)$ versus the rescaled noise parameter of the system, $|q-q_c|L^{1/\nu }$. These plots reveal the respective data collapse of the functions corresponding to different system sizes into the expected universal scaling functions $\widetilde{M}$, $\widetilde{\chi }$ and $\widetilde{U}$ when using the exponents from Table 2. In Fig. 10, we present data collapses for the *p* values in regular and small-world regime $p=0$, $p=10^{-4}$, and $p=10^{-3}$, respectively. As seen in the figures, changing the rewiring probability *p* exhibits similar behavior for the data collapse of each quantity, regarded that we use the suitable exponents from Table 2 for each rewiring probability *p*. The universal behavior for each rescaled function, where different system sizes all collapse in one line, indicates that the critical exponents obtained for the system present reasonable precision in the small-world regime, where $0< p < 0.01$^38^.

We conclude that the disorder promoted by long-range small-world interactions on square lattices strongly improves the magnetic order, remaining more robust under the social temperature parameter *q*. The rewiring probability also changes the critical behavior of the three-state majority-vote model, while leaving the average connectivity $\langle k\rangle = 4$ unchanged.

## Concluding remarks

We investigate the statistical mechanics of the three-state majority-vote model on small-world networks. Similar to the isotropic two-state version, the opinion variable of an individual $\sigma$ may assume only one of three discrete values, such as $\sigma = {1, 2, 3}$. In this model, an individual tends to adopt the opinion of the majority of its neighbors with probability $(1 - q)$, and a different opinion with a probability *q*. Here, *q* stands for the noise parameter of the model and it induces the social disorder. From the generalized update rules for an opinion given by Eqs. (1), the three-state majority-vote model it is placed within the framework of previous studies on other complex network structures, as the square lattice^12,13,32^, random graphs^14^, and Barabási-Albert (scale-free) networks^39^.

Small-world networks are built starting with two dimensional square lattices whose links are rewired with probability *p*, with double links forbidden. We explore the main topological characteristics of these networks by means of degree distributions, clustering coefficient and average path length calculations, which reveal the role of disorder in the shrinking of the networks. We show that the average clustering coefficient $C_L(p)$ reaches a maximum for $p \sim O(1)$, and the average shortest path length $\ell _L(p)$ decreases in the presence of rewired links as expected. These results indicate the emergence of the small-world effect in the networks considered in this work.

Monte Carlo simulations and finite-size scaling analysis reveal that the system exhibits second-order phase transitions, spontaneous symmetry breaking and long-range correlations. The critical noise parameter, $q_c(p)$ is a monotonically increasing function of *p*, thereby suggesting that randomness in the topology of the network makes the ordered opinion phase more resilient against social thermal fluctuations induced by *q*. We estimate the critical exponents for different values of the rewiring probability *p*, and confirm them by means of the data-collapse of the average magnetization, magnetic susceptibility, and fourth-order Binder cumulant into their universal scaling functions. For $p=0$, the two-dimensional regular lattice with periodic boundary conditions obtains and the critical exponent values measured are in agreement with numerical calculations in the literature, consistent with the three-state Potts model. However, in the presence of topological disorder $p>0$, the results suggest that the critical exponents depend on the value of the rewiring probability *p*; thus indicating they belong to different universality classes.

The numerical results suggest that the link-rewiring scheme and the ensuing topological disorder effects slightly increase the effective critical dimension of the system above $d=2$, a manifestation of the fact that the network changes its length scales due to the small-world property. This result agrees with previous studies, where the effective dimension of the complex networks, in general, does not agree with the spatial dimension of the embedding lattice.
